# Occupational motions such as kneeling and squatting are associated with the increased development of medial meniscus posterior root tears, regardless of the medial posterior tibial slope angle

**DOI:** 10.1002/jeo2.70276

**Published:** 2025-05-19

**Authors:** Koki Kawada, Yusuke Yokoyama, Masanori Tamura, Yuki Okazaki, Toshifumi Ozaki, Takayuki Furumatsu

**Affiliations:** ^1^ Department of Orthopaedic Surgery Okayama University Graduate School of Medicine, Dentistry, and Pharmaceutical Sciences Okayama Japan; ^2^ Department of Orthopaedic Surgery Japanese Red Cross Okayama Hospital Okayama Japan

**Keywords:** kneeling, meniscus, occupational motion, posterior root tear, posterior tibial slope

## Abstract

**Purpose:**

The relationship between occupational motions and the medial posterior tibial slope (MPTS) with the development of medial meniscus posterior root tears (MMPRTs) has not been investigated. The development of non‐traumatic degenerative MMPRTs may be influenced by repetitive occupational motions and bone morphological characteristics. Herein, we examined the association between occupational motions and MPTS in patients with MMPRT development.

**Methods:**

During the first medical examination, MPTS was measured using lateral knee radiographic images, and occupational motions were investigated in 559 patients (591 knees). Occupational motions were classified as kneeling and squatting, standing and walking, sitting, lifting heavy weights, and housework. Mann–Whitney *U* test was used to compare patient characteristics between male and female patients and MPTS relative to occupational motion.

**Results:**

The most frequent occupational motion was housework (160/559 patients, 28.6%), followed by kneeling and squatting (140/559, 25.0%), standing and walking (128/559, 22.9%), sitting (82/559, 14.7%), and lifting heavy weights (49/559, 8.8%). Furthermore, housework (10.0 ± 2.6°) involved significantly greater MPTS than kneeling and squatting (9.3 ± 2.7°; *p* = 0.012). However, the MPTS associated with other occupational motions was not significantly different from that associated with housework.

**Conclusion:**

The most frequent occupational motion among patients with MMPRTs was housework, followed by kneeling and squatting. Patients who performed housework tended to have a higher MPTS. Occupational motions such as kneeling and squatting potentially increase the development of MMPRTs, even without a high MPTS.

**Level of Evidence:**

Level IV.

**Abbreviations**
BMI: body mass index
MM: medial meniscus
MMPRT: medial meniscus posterior root tear
MPTS: medial posterior tibial slope
OA: osteoarthritis

## INTRODUCTION

There are two types of medial meniscus (MM) posterior root tears (MMPRTs): traumatic MMPRTs, which are more common in highly active young patients, and degenerative MMPRTs, which occur during routine activities in middle‐aged and older patients [3, 9, 23]. Risk factors for the development of degenerative MMPRTs include varus lower limb alignment, high body mass index (BMI), and female sex [13, 14, 36]. Recently, bone morphological characteristics such as a large medial posterior tibial slope (MPTS) and narrow intercondylar notch have also been reported as risk factors for the development of degenerative MMPRTs [1, 8, 12, 27].

In particular, MPTS has received much attention, as it has been reported to not only lead to MMPRTs but also to affect postoperative outcomes after the repair of MMPRTs [6, 28]. MPTS is reportedly affected by various factors such as ethnic origin, age and sex, but there is no consensus yet [16]. Concerning the sex differences in the MPTS, there are reports that the differences are larger for females [5], whereas others report that there are no sex differences [30]. In addition, concerning the effect of age on MPTS, it has been reported that MPTS decreases with age [32].

Also, repetitive occupational motions, particularly kneeling and squatting, are associated with knee osteoarthritis (OA) [25]. Occupational kneeling and squatting are associated with degeneration of the MM [29] and are often considered occupation‐related disorders [2]. However, to the best of our knowledge, the relationship between MMPRTs and occupational motions has not been investigated. Therefore, in this study, we aimed to examine the association between occupational motions and MMPRT development. We also aimed to examine the association between occupational motions and MPTS in patients with MMPRTs. We hypothesized that occupational motions involving kneeling and squatting are strongly associated with the development of MMPRTs and that these occupational motions are also associated with a greater MPTS.

## MATERIALS AND METHODS

### Patients

This retrospective study was approved by the Ethics Committee of our university and was performed in line with the principles of the Declaration of Helsinki. Written informed consent was obtained from all patients.

The study involved 610 knees of 578 patients who underwent pullout repair for MMPRTs between January 2018 and December 2023 at our institution. Of these, 8 knees of eight patients who had previously undergone ipsilateral knee surgery, 10 knees of 10 patients with anterior cruciate ligament insufficiency or injury and 1 knee of one patient with multiple ligament injury were excluded, and the remaining 591 knees of 559 patients were subjected to the final review.

### Radiographic assessments

**Figure 1:**
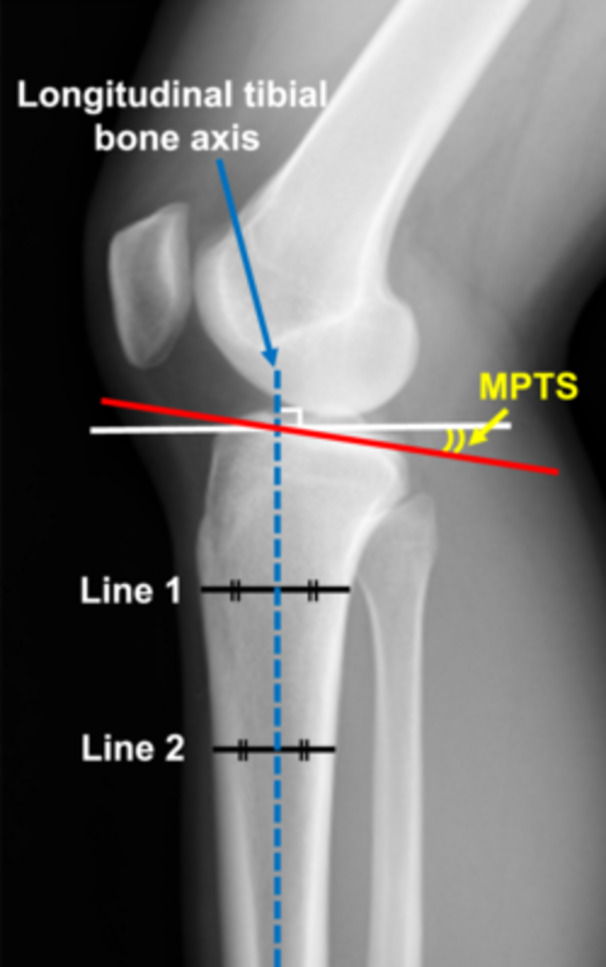
Measurement methods of MPTS. MPTS was defined as the angle between the line perpendicular to the tibial bone axis (white line) and the medial tibial plateau (red line). The longitudinal tibial bone axis (blue dotted line) was defined by the line created by connecting the midpoint of the anteroposterior diameter of the tibia just inferior to the tibial tubercle (Line 1) and the midpoint of the anteroposterior diameter of the tibial shaft 5 cm distal from Line 1 (Line 2). MPTS, medial posterior tibial slope.

MPTS was measured using radiographic images of the lateral knee joint in the non‐weight‐bearing position [11]. The radiographic images of the lateral knee joint were unified so that the posterior condyles of the femur overlapped [33]. MPTS was defined as the angle between the line perpendicular to the tibial bone axis and the medial tibial plateau (Figure 1). The longitudinal tibial bone axis was defined by the line created by connecting the midpoint of the anteroposterior diameter of the tibia just inferior to the tibial tubercle (Line 1) and the midpoint of the anteroposterior diameter of the tibial shaft 5 cm distal from Line 1 (Line 2).

### Occupational motions in patients with MMPRTs

During the first medical examination, orthopaedic surgeons investigated the patients' occupational motions by asking them the following questions: ‘What is your primary occupation at present or in the past?’ and ‘What is the primary motion or posture in your occupation?’ Patients with multiple occupational motions were determined by asking them about the occupational motion that they engaged in most regularly.

Occupational motions were classified into five categories: kneeling and squatting, standing and walking, sitting, lifting heavy weights, and housework. Kneeling and squatting were mainly performed by farm and childcare workers; standing and walking were mainly performed by cooks and hospitality workers; sitting was mainly performed by office and desk workers; lifting heavy weights was mainly performed by delivery workers, manual labourers, and construction workers; and housework was mainly performed by homemakers and unemployed individuals. The classification of occupational motions was determined by asking patients about their work and the most common motions during the first medical examination by orthopaedic surgeons. The details of the first medical examination were recorded and summarized in the electronic medical records by the orthopaedic surgeons. This study retrospectively investigated the content of these records.

### Statistical analysis

Statistical analysis was performed using the EZR software (Saitama Medical Center, Japan). Shapiro–Wilk test was used to evaluate the normality of the distribution of the data, and all patient characteristics were non‐normally distributed. Comparison of patient characteristics between male and female patients and comparison of MPTS in relation to occupational motions were performed using the Mann–Whitney *U* test.

MPTS measurements were obtained by two orthopaedic surgeons at two separate time points 6 weeks apart to assess intra‐ and inter‐observer reliability. In addition, a power analysis of the Mann–Whitney *U* test for MPTS between the kneeling and squatting versus housework groups was performed using G Power (University of Düsseldorf).

## RESULTS

The patient characteristics are shown in Table 1.

**Table 1:** Patient characteristics.

	All patients (*n* = 559)	Male patients (*n* = 113)	Female patients (*n* = 446)	*p* b
Age (years)	65.5 ± 8.9	64.2 ± 10.0	65.9 ± 8.6	0.196
Body weight (kg)	63.7 ± 13.5	76.5 ± 14.8	60.5 ± 11.0	<0.001*
Body height (m)	1.57 ± 0.08	1.67 ± 0.07	1.54 ± 0.06	<0.001*
BMI (kg/m^2^)	25.8 ± 4.3	27.2 ± 4.3	25.5 ± 4.2	<0.001*
Injury onset‐to‐operation time (days)^a^	74.5 ± 93.1	87.9 ± 118.5	71.0 ± 85.1	0.116
MPTS (°)	9.7 ± 2.8	9.4 ± 3.2	9.8 ± 2.7	0.250

*Note*: Values are presented as mean ± standard deviation. Statistical analysis was performed using the Mann–Whitney *U* test.

Abbreviations: BMI, body mass index; MPTS, medial posterior tibial slope.

^a^ This value was obtained for 543 knees, after excluding 48 knees in which the injury onset was unknown, among 591 knees of 559 patients.

^b^ Comparison between male and female patients.

* *p* < 0.05.

**Figure 2:**
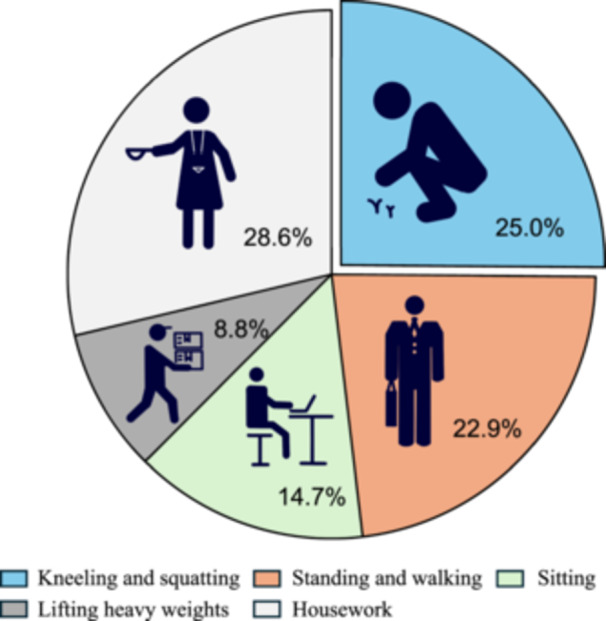
Occupational motions in patients with MMPRTs. The figure presents schematic illustrations of each occupational motion and the corresponding percentages in the pie chart. MMPRTs, medial meniscus posterior root tears.

**Figure 3:**
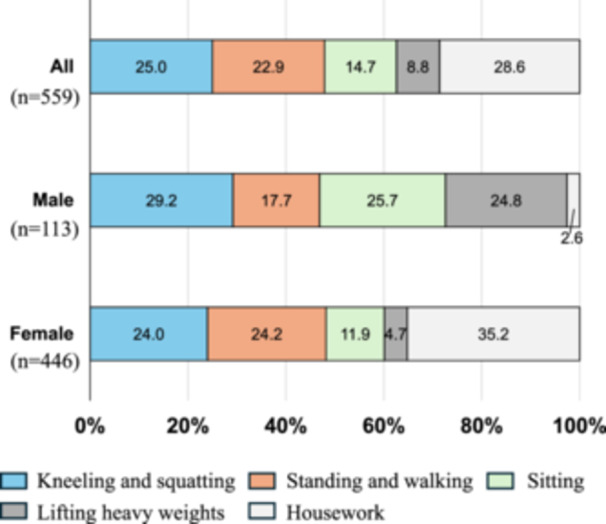
Occupational motions in male and female patients with MMPRTs. The percentage of patients performing each occupational motion is shown in the bar graph. MMPRTs, medial meniscus posterior root tears.

The most frequent occupational motion was housework (160/559 patients, 28.6%), followed by kneeling and squatting (140/559 patients, 25.0%), standing and walking (128/559 patients, 22.9%), sitting (82/559 patients, 14.7%), and lifting heavy weights (49/559 patients, 8.8%; Figure 2). In male patients, the most frequent occupational motions were kneeling and squatting (33/113 patients, 29.2%), followed by sitting (29/113 patients, 25.7%) and lifting heavy weights (28/113 patients, 24.8%; Figure 3). In female patients, the most frequent occupational motion was housework (157/446 patients, 35.2%), followed by standing and walking (108/446 patients, 24.2%), and kneeling and squatting (107/446 patients, 24.0%; Figure 3).

The MPTS scores for each type of occupational motion are presented in Table 2. In the comparison of MPTS among occupational motions, the MPTS associated with housework was significantly greater than that associated with kneeling or squatting (*p* = 0.012; Table 3).

**Table 2:** MPTS for each occupational motion.

Occupational motion	MPTS (°)
Kneeling and squatting	9.3 ± 2.7
Standing and walking	9.8 ± 2.9
Sitting	9.8 ± 3.2
Lifting heavy weights	9.7 ± 3.1
Housework	10.0 ± 2.6

*Note*: Values are presented as mean ± standard deviation.

Abbreviation: MPTS, medial posterior tibial slope.

**Table 3:** Comparison of MPTS between occupational motions.

Occupational motion	Kneeling and squatting	Standing and walking	Sitting	Lifting heavy weights	Housework
Kneeling and squatting	–	0.218	0.213	0.412	0.012*
Standing and walking	–	–	0.833	0.951	0.256
Sitting	–	–	–	0.998	0.482
Lifting heavy weights	–	–	–	–	0.458
Housework	–	–	–	–	–

*Note*: Statistical analysis was performed using the Mann–Whitney *U* test.

Abbreviations: MPTS, medial posterior tibial slope.

* *p* < 0.05.

The intra‐ and inter‐observer reliability values of the MPTS measurements were 0.967 and 0.928, respectively. The actual power of the Mann–Whitney *U* test for comparison of MPTS between the kneeling and squatting (sample size = 137) and housework (sample size = 163) groups was 70.3% (effect size = 0.29; *α* error = 0.05).

## DISCUSSION

The most important finding of this study, which included a large group of patients with MMPRTs (*n* = 559), was that housework was the most frequent occupational motion in patients with MMPRTs, followed by kneeling and squatting. Moreover, the MPTS associated with housework was significantly greater than that associated with kneeling and squatting.

The fact that patients performing housework were the most numerous was contrary to expectations, as these motions seemed to have the lowest risk and smallest percentage. There are several possible reasons for this finding. First, the proportion of female patients with MMPRTs is quite high, and housework may account for a large proportion of occupational motions in female patients. The incidence of MMPRTs has been reported to be approximately three to four times higher in female individuals [4, 15, 19]. In this study, the number of females with MMPRTs was four times higher than that of men. Second, patients whose occupational motions consisted of housework tended to have a greater MPTS. A large MPTS has already been reported to be associated with the development of MMPRTs [21]. A larger MPTS has also been reported to be associated with the development of MMPRTs at a younger age [17]. In this study, in addition to the high proportion of females among the patients with MMPRTs, the high MPTS of those who did housework may have contributed to the high number of patients with MMPRTs who performed housework.

In this study, after housework, the next most frequent occupational motion involved kneeling and squatting. The normal knee joint is known to exhibit a lateral pivot motion during walking [18] and a medial pivot motion during knee flexion movements, such as kneeling and squatting [24]. In the squatting motion, the femur moves 25 mm posteriorly with respect to the tibia [34]. Clinically, kneeling and squatting motions have been reported to be associated with knee OA and meniscal injuries [2, 25, 29]. However, to the best of our knowledge, the association between kneeling and squatting motions and MMPRTs has not been clinically reported. Melugin et al. reported that the medial shear and traction forces at the posterior root of the MM increased as the knee flexion angle increased [21]. Walker et al. reported that the posterior shear forces on the posterior root of the MM increased after the knee joint was flexed by >30° [34]. In this study, the only significant difference in MPTS between housework and other occupational motions was observed in kneeling and squatting. This result suggests that kneeling and squatting may be involved in the development of MMPRT regardless of MPTS compared to other occupational motions.

MPTS has been noted to influence tibial translation, the strain on the native or grafted cruciate ligament, and the pressure distribution on the cartilage [20]. In recent years, MPTS has attracted particular attention as one of the important bone morphological characteristics that affect the development of MMPRTs and the outcomes after treatment for MMPRTs [6, 28]. Reports indicate that MPTS decreases with age, but there is currently no consensus [16, 32]. As a second hypothesis of this study, we stated that MPTS would be greater in patients with MMPRTs who had kneeling and squatting as occupational motions. However, in this study, we also investigated the relationship between MPTS and occupational motions, and the results showed that MPTS was greatest in patients with MMPRTs who had housework as an occupational activity. However, we consider that this does not mean that higher MPTS is more likely to be acquired in people who engage in housework as an occupational motion, but it does suggest that even people who engage in housework as an occupational motion may be more likely to develop MMPRTs if they have a high MPTS.

The relationship observed in this study between MPTS and occupational motion in patients with MMPRTs has clinical relevance, as individuals who kneel and squat regardless of MPTS need to be cautious about the risk of developing MMPRTs. Moreover, even individuals involved in housework need to be more cautious if their MPTS is high.

The onset of MMPRTs presents a high rate of painful popping episodes, which are characterized by sudden posteromedial knee pain [10]. Furumatsu et al. reported that the onset of MMPRTs occurred while descending steps or walking on level ground in more than half of the cases and during squatting motions such as knee flexion in a relatively low percentage (13%) of cases [10]. In a finite element analysis, Yokoe et al. reported that the descending step motion applies twice as much force to the posterior root of the MM as walking and an equivalent force as jogging [35]. In this study, we also asked about the sports activities of patients with MMPRTs, but only 80 out of 559 patients (14.3%) engaged in regular sports activities. As a result, this study was unable to consider the relationship between occupational motions, sports activities, and bone morphological characteristics. In addition to occupational motions, further research is needed regarding the association of sports and daily activities with the development of MMPRTs.

Reports indicate that a high BMI is associated with the development of MMPRTs [14, 31]. In this study, males had a higher BMI than females. In addition, males had the highest proportion of kneeling and squatting in this study, whereas, in females, housework had the highest proportion, with kneeling and squatting being the third most common. Even for the same occupational motions, the magnitude of the load on the knee joint may differ with BMI [7]. Therefore, the influence of BMI should be considered when further examining occupational motions, sports, and daily activities in relation to the development of MMPRTs.

The injury onset‐to‐operation time is an important clinical factor in MMPRTs, and it is known that earlier repair after injury leads to better postoperative clinical outcomes [22, 26]. In this study, there were no significant differences in the injury onset‐to‐operation time between the sexes or occupational motions. Therefore, in this study, which examined occupational motions and MPTS in the development of MMPRTs, the injury onset‐to‐operation time may not have been an important factor.

This study had certain limitations. First, it was a retrospective study. However, all patients included in this study were able to confirm their occupational motions. Second, the sample size was insufficient to compare MPTS among the occupational motions. In the post‐hoc analysis, the actual power of the Mann–Whitney *U* test for the MPTS between the kneeling and squatting versus housework groups was 70.3%, which was less than 80%. Therefore, additional studies with larger samples are required. Third, the exposure periods for the main occupational motions could not be evaluated. The exposure period for occupational motions may vary among patients. The duration of occupational motions should also be investigated in future studies. Fourth, the proportion of general occupational movements is unknown and may vary by region. Future cohort studies involving regional populations, including patients without MMPRTs, are required. Fifth, the classification of occupational motions was determined by orthopaedic surgeons asking patients during the first medical examination, and the evaluation was not conducted by multiple examiners. In addition, the criteria for defining occupational motions were subjective and based on a single question. Sixth, even among patients with occupational motions related to housework, it is possible that there were large differences in regular sports activities and the amount of movement, but these were not evaluated. In the future, it will be necessary to investigate the background of patients with MMPRTs and to determine factors such as sports activities, the amount of movement, and the exposure time of occupational motions.

## CONCLUSIONS

Our findings showed that the most frequent occupational motion among patients with MMPRTs was housework, followed by kneeling and squatting. Patients who perform housework tend to have a higher MPTS. Occupational motions such as kneeling and squatting potentially increase the development of MMPRTs, even without a high MPTS.

## AUTHOR CONTRIBUTIONS

Takayuki Furumatsu and Koki Kawada conceptualized this study and performed the documentation. All authors performed data collection and analysis. All authors commented on the first draft of the manuscript and approved the final draft.

## CONFLICT OF INTEREST STATEMENT

The authors declare no conflicts of interest.

## ETHICS STATEMENT

This study was performed in accordance with the principles of the Declaration of Helsinki. Approval was granted by the Ethics Committee of Okayama University (No. 1857). Written informed consent was obtained from all patients.

## Data Availability

The data that support the findings of this study are available from the corresponding author upon reasonable request.

## References

[1] Altinayak H , Karatekin YS . Increased medial femoral condyle angle and narrow intercondylar notch are associated with medial meniscus posterior root tear. Arthroscopy. 2023;39:2154–2163. 36868529 10.1016/j.arthro.2023.02.020

[2] Bahns C , Bolm‐Audorff U , Seidler A , Romero Starke K , Ochsmann E . Occupational risk factors for meniscal lesions: a systematic review and meta‐analysis. BMC Musculoskelet Disord. 2021;22:1042. 34911509 10.1186/s12891-021-04900-7 PMC8672613

[3] Bogas Droy H , Dardenne T , Djebara A , Pujol N . Long‐term clinical and radiological outcomes after arthroscopic partial meniscectomy on stable knees are better for traumatic tears when compared to degenerative lesions: a systematic review. Knee Surg Sports Traumatol Arthrosc. 2025;33:107–123. 39031666 10.1002/ksa.12329

[4] Chang PS , Radtke L , Ward P , Brophy RH . Midterm outcomes of posterior medial meniscus root tear repair: a systematic review. Am J Sports Med. 2022;50:545–553. 33780278 10.1177/0363546521998297

[5] Chen Y , Ding J , Dai S , Yang J , Wang M , Tian T , et al. Radiographic measurement of the posterior tibial slope in normal Chinese adults: a retrospective cohort study. BMC Musculoskelet Disord. 2022;23:386. 35473639 10.1186/s12891-022-05319-4 PMC9040249

[6] Choi NH . Editorial commentary: determinants of outcome after repair of knee medial meniscus posterior root tear are multifactorial. Arthroscopy. 2023;39:1384–1385. 37147070 10.1016/j.arthro.2022.12.017

[7] Collins AT , Kulvaranon M , Spritzer CE , McNulty AL , DeFrate LE . The influence of obesity and meniscal coverage on in vivo tibial cartilage thickness and strain. Orthop J Sports Med. 2020;8:2325967120964468. 33330731 10.1177/2325967120964468 PMC7720327

[8] Dzidzishvili L , Allende F , Allahabadi S , Mowers CC , Cotter EJ , Chahla J . Increased posterior tibial slope is associated with increased risk of meniscal root tears: a systematic review. Am J Sports Med. 2024;52:3427–3435. 38362610 10.1177/03635465231225981

[9] Dzidzishvili L , Ostojic M , Chahla J . The journey from silent epidemic to solved mystery: where we stand and the path forward in meniscal root preservation surgery. Knee Surg Sports Traumatol Arthrosc. 2024;33:789–792. 39435620 10.1002/ksa.12520

[10] Furumatsu T , Okazaki Y , Okazaki Y , Hino T , Kamatsuki Y , Masuda S , et al. Injury patterns of medial meniscus posterior root tears. Orthopa Traumatol Surg Res. 2019;105:107–111. 10.1016/j.otsr.2018.10.001 30442555

[11] Hiranaka T , Furumatsu T , Okazaki Y , Yamawaki T , Okazaki Y , Kodama Y , et al. Steep medial tibial slope and prolonged delay to surgery are associated with bilateral medial meniscus posterior root tear. Knee Surg Sports Traumatol Arthrosc. 2021;29:1052–1057. 32488369 10.1007/s00167-020-06079-1

[12] Hiranaka T , Furumatsu T , Yokoyama Y , Higashihara N , Tamura M , Kawada K , et al. Intercondylar notch width and osteophyte width impact meniscal healing and clinical outcomes following transtibial pullout repair of medial meniscus posterior root tears. Knee Surg Sports Traumatol Arthrosc. 2024;32:116–123. 38226691 10.1002/ksa.12032

[13] Hiranaka T , Furumatsu T , Yokoyama Y , Higashihara N , Tamura M , Kawada K , et al. Weight loss enhances meniscal healing following transtibial pullout repair for medial meniscus posterior root tears. Knee Surg Sports Traumatol Arthrosc. 2024;32:143–150. 38226719 10.1002/ksa.12037

[14] Hwang BY , Kim SJ , Lee SW , Lee HE , Lee CK , Hunter DJ , et al. Risk factors for medial meniscus posterior root tear. Am J Sports Med. 2012;40:1606–1610. 22582224 10.1177/0363546512447792

[15] Kamatsuki Y , Furumatsu T , Hiranaka T , Okazaki Y , Kintaka K , Kodama Y , et al. Epidemiological features of acute medial meniscus posterior root tears. Int Orthop. 2023;47:2537–2545. 37329453 10.1007/s00264-023-05848-0 PMC10522759

[16] Klein C , Rahab R , Rouanet T , Deroussen F , Demester J , Gouron R . Is an excessively high posterior tibial slope a predisposition to knee injuries in children? Systematic review of the literature. Orthop Traumatol Surg Res. 2024. Online ahead of print. 10.1016/j.otsr.2024.104033 39488241

[17] Kodama Y , Furumatsu T , Tamura M , Okazaki Y , Hiranaka T , Kamatsuki Y , et al. Steep posterior slope of the medial tibial plateau and anterior cruciate ligament degeneration contribute to medial meniscus posterior root tears in young patients. Knee Surg Sports Traumatol Arthrosc. 2023;31:279–285. 35978177 10.1007/s00167-022-07095-z

[18] Koo S , Andriacchi TP . The knee joint center of rotation is predominantly on the lateral side during normal walking. J Biomech. 2008;41:1269–1273. 18313060 10.1016/j.jbiomech.2008.01.013 PMC2481385

[19] Lin L , Jiang S , Yang S , Yang G , Xie B , Zhang L . Identical clinical outcomes between neutral and classic targeted alignments after high tibial osteotomy in medial meniscus posterior root tear: a prospective randomized study. Int Orthop. 2024;48:427–437. 37676496 10.1007/s00264-023-05960-1

[20] Mabrouk A , Chou TA , Duouguih W , Onishi S , Mansour A , Ollivier M . Medial posterior tibial slope measurements are overestimated on long radiographs and 3D CT compared to measurements on short lateral radiographs. J Exp Orthop. 2024;11:e70120. 39697993 10.1002/jeo2.70120 PMC11653213

[21] Melugin HP , Brown JR , Hollenbeck JFM , Fossum BW , Whalen RJ , Ganokroj P , et al. Increased posterior tibial slope increases force on the posterior medial meniscus root. Am J Sports Med. 2023;51:3197–3203. 37715505 10.1177/03635465231195841

[22] Moon HS , Choi CH , Jung M , Lee DY , Hong SP , Kim SH . Early surgical repair of medial meniscus posterior root tear minimizes the progression of meniscal extrusion: 2‐year follow‐up of clinical and radiographic parameters after arthroscopic transtibial pull‐out repair. Am J Sports Med. 2020;48:2692–2702. 32730732 10.1177/0363546520940715

[23] Mundal K , Geeslin AG , Solheim E , Inderhaug E . Differences between traumatic and degenerative medial meniscus posterior root tears: a systematic review. Am J Sports Med. 2025;53:228–233. 38600780 10.1177/03635465241237254 PMC11689786

[24] Murakami K , Hamai S , Okazaki K , Ikebe S , Shimoto T , Hara D , et al. In vivo kinematics of healthy male knees during squat and golf swing using image‐matching techniques. Knee. 2016;23:221–226. 26783190 10.1016/j.knee.2015.08.004

[25] Muraki S , Oka H , Akune T , En‐yo Y , Yoshida M , Nakamura K , et al. Association of occupational activity with joint space narrowing and osteophytosis in the medial compartment of the knee: the ROAD study (OAC5914R2). Osteoarthritis Cartilage. 2011;19:840–846. 21447396 10.1016/j.joca.2011.03.008

[26] Nie S , Li H , Liao X , Liu Q , Lan M . Younger patients, lower BMI, complete meniscus root healing, lower HKA degree and shorter preoperative symptom duration were the independent risk factors correlated with the good correction of MME in patients with repaired MMPRTs. Knee Surg Sports Traumatol Arthrosc. 2023;31:3775–3783. 36790456 10.1007/s00167-023-07330-1

[27] Oeding JF , Dean MC , Hevesi M , Chahla J , Krych AJ . Steeper slope of the medial tibial plateau, greater varus alignment, and narrower intercondylar distance and notch width increase risk for medial meniscus posterior root tears: a systematic review. Arthroscopy. 2024. Online ahead of print. 10.1016/j.arthro.2024.10.031 39505159

[28] Okazaki Y , Furumatsu T , Kodama Y , Kamatsuki Y , Okazaki Y , Hiranaka T , et al. Steep posterior slope and shallow concave shape of the medial tibial plateau are risk factors for medial meniscus posterior root tears. Knee Surg Sports Traumatol Arthrosc. 2021;29:44–50. 31243503 10.1007/s00167-019-05590-4

[29] Rytter S , Jensen LK , Bonde JP , Jurik AG , Egund N . Occupational kneeling and meniscal tears: a magnetic resonance imaging study in floor layers. J Rheumatol. 2009;36:1512–1519. 19411395 10.3899/jrheum.081150

[30] Shin CH , Syed AN , Swanson ME , Kushare IV , Shea KG , Ganley TJ , et al. Evaluation of tibial slope on radiographs in pediatric patients with tibial spine fractures: an age‐ and sex‐matched study. Orthop J Sports Med. 2024;12:23259671241256445. 39100212 10.1177/23259671241256445 PMC11295229

[31] Siller RL , Raja H , Lindeman RW . Association of posterior horn meniscus tears with obesity: a retrospective study. J Knee Surg. 2025;38:1–6. 38295833 10.1055/s-0044-1779512

[32] Singh S , Chaurasia A , Shantanu K , Mohan R , Chaudhary S , Kumar D , et al. Anatomical variations in the posterior tibial slope in the North Indian population: a hospital‐based study. Cureus. 2023;15(7):e41338. 37546042 10.7759/cureus.41338 PMC10397523

[33] Vieider RP , Mehl J , Rab P , Brunner M , Schulz P , Rupp MC , et al. Malrotated lateral knee radiographs do not allow for a proper assessment of medial or lateral posterior tibial slope. Knee Surg Sports Traumatol Arthrosc. 2024;32:1462–1469. 38629758 10.1002/ksa.12170

[34] Walker PS , Arno S , Bell C , Salvadore G , Borukhov I , Oh C . Function of the medial meniscus in force transmission and stability. J Biomech. 2015;48:1383–1388. 25888013 10.1016/j.jbiomech.2015.02.055

[35] Yokoe T , Ouchi K , Yamaguchi Y , Enzaki M , Tajima T , Chosa E . Shear stress in the medial meniscus posterior root during daily activities. Knee. 2023;43:176–183. 37441878 10.1016/j.knee.2023.06.008

[36] Zhan H , Liu Z , Wang Y , Chen Y , Teng F , Yang A , et al. Radiographic OA, bone marrow lesions, higher body mass index and medial meniscal root tears are significantly associated with medial meniscus extrusion with OA or medial meniscal tears: a systematic review and meta‐analysis. Knee Surg Sports Traumatol Arthrosc. 2023;31:3420–3433. 37099153 10.1007/s00167-023-07418-8

